# Promoting COVID-19 Vaccination Using the Health Belief Model: Does Information Acquisition from Divergent Sources Make a Difference?

**DOI:** 10.3390/ijerph19073887

**Published:** 2022-03-24

**Authors:** Xiaodong Yang, Lai Wei, Zhiyue Liu

**Affiliations:** School of Journalism and Communication, Shandong University, Jinan 250100, China; xyang012@e.ntu.edu.sg (X.Y.); weilai@mail.sdu.edu.cn (L.W.)

**Keywords:** COVID-19 pandemic, information acquisition, vaccination intention, health belief model

## Abstract

As a promising approach to stop the escalation of the pandemic, COVID-19 vaccine promotion is becoming a challenging task for authorities worldwide. The purpose of this study was to identify the effective sources for disseminating information on the COVID-19 vaccine to promote individuals’ behavioral intention to take the vaccine. Based on the Health Belief Model (HBM), this study illustrated the mechanism of how COVID-19 information acquisition from different sources was transformed into vaccination intentions via health beliefs. Using an online survey in China, the structural equation model results revealed that perceived benefits and cues to action were positively associated with COVID-19 vaccination intentions, and perceived barriers were negatively related to the intentions. However, perceived susceptibility and perceived severity had no significant relationships with the intentions. Moreover, the findings unveiled differences in the effects of acquiring information via multiple sources among traditional media, new media, and interpersonal interactions. Notably, new media and interpersonal interactions were more salient in promoting vaccination intention via health beliefs, compared with traditional media. The findings from this study will benefit health officials in terms of utilizing different information sources in vaccine programs.

## 1. Introduction

With dramatic health, social and economic impacts, the COVID-19 pandemic has progressed into a public health crisis worldwide. By protecting people from the virus, COVID-19 vaccination is becoming a promising approach to stop the escalation of the pandemic. Despite tremendous vaccination effort and investment, vaccine hesitancy or refusal remains as one of the major hindrances to an effective COVID-19 vaccine program. For instance, low COVID-19 vaccine acceptance rates were more salient in the Middle East, Eastern Europe and Russia [1]. It was reported that a COVID-19 vaccine acceptance rate below 60% would hinder successful control of the pandemic. The authorities face the challenging task of promoting public acceptance of COVID-19 vaccination. Extensive studies on health risk communication have accentuated the role of information acquisition in health behavior promotion [2,3,4,5]. The nature of COVID-19 as an emerging infectious disease has raised the demand for communication-based interventions, as a lack of information about the pandemic could lead to numerous negative outcomes, such as spreading fears and vaccine skepticism [6,7,8]. As such, information dissemination about the COVID-19 vaccine is foundational to promoting vaccination intention. Previous literature on vaccines suggests that exposure to health information through divergent sources has both positive and negative effects on vaccination [9,10]. Hence, identifying the most effective information sources for increasing individuals’ intention to take the COVID-19 vaccine is important for the success of the vaccine program.

Particularly, existing studies on health communication have suggested that communication factors may not influence the public directly [11]. Instead, much work on health behavior promotion has paid particular attention to explore the indirect paths through which communication factors have effects on the public [12,13]. Notably, scholars have heightened the need for figuring out the indirect paths via psychological factors [14,15]. Thus, this study aimed to examine how individuals’ acquisition of information about the COVID-19 vaccine from divergent sources is related to their vaccination intention by affecting psychological factors.

Focusing on psychological factors such the perceived risk of health problems, the perceived benefits of action and barriers to action, and cues triggering action, the Health Belief Model (HBM) has been one of the most widely used models for examining the determinants of individuals’ intentions to take a vaccine [16,17]. However, very few studies have examined the factors that contribute to health beliefs. To fill this research gap, this study proposed to examine the contributions of individuals’ health information acquisition to their health beliefs. Particularly, this study applied the HBM as the theoretical underpinning to investigate how individuals’ acquisition of information on the COVID-19 vaccine is related to their intention to take the COVID-19 vaccine through enhancing health beliefs.

Overall, the purpose of the present study was twofold. Firstly, based on the Health Belief Model (HBM), this study aimed to illustrate the mechanism underlying how COVID-19 information acquisition is transformed into vaccination intentions. Secondly, considering the distinctive roles of divergent information sources, we sought to identify the most advantageous sources of information in promoting COVID-19 vaccination. Findings from this study will not only theoretically contribute to the literature on vaccination, but will also practically benefit the authorities in terms of locating efficacious information sources for delivering health information during outbreaks of public health crises.

## 2. Theoretical Model and Development of Hypotheses

### 2.1. Health Belief Model

The Health Belief Model (HBM) was initially developed to explain the failure of individuals to participate in health promotion programs [18,19]. According to the HBM, individuals’ intentions to adopt health actions are determined by their perceptions of susceptibility, severity, benefits, barriers and cues to action [20]. Empirically, the HBM has been one of the most widely used conceptual frameworks in health behavior studies. A significant body of literature has demonstrated the effectiveness of the HBM in various health contexts, such as promoting cancer-related examinations [21], health risk management [22] and vaccination uptake [23]. Besides, prior research has applied various models to explain vaccination intentions, including the theory of planned behavior (TPB). Compared with the TPB, we chose the HBM as the model for the current study, because the HBM was specifically developed for focusing on preventive health research [23,24,25]. Overall, this model offers an ideal framework for promoting COVID-19 vaccination.

***Perceived susceptibility.*** In particular, perceived susceptibility refers to beliefs about the probability of contracting a disease. Many scholars have demonstrated that people with higher levels of perceived susceptibility are more likely to engage in health behaviors to decrease the risk, compared with those who have less perceived susceptibility. For example, Orji et al. [26] reported that perceived susceptibility was a significant determinant of healthy eating behavior. Likewise, Guidry et al. [27] revealed the direct effect of perceived susceptibility on Zika vaccine uptake intent. In light of these empirical findings, this study proposed the following hypothesis:

**H1.**  *Perceived susceptibility is positively associated with behavioral intention to take the COVID-19 vaccine*.

***Perceived Severity.*** Defined as one’s concern over the seriousness of health issues and the related clinical and social consequences, perceived severity is also a salient factor predicting health behaviors. For instance, a study of avian influenza reported that perceived severity had significant impacts on individuals’ preventive health actions [28]. Another study on H1N1 also documented the critical role of perceived severity in promoting individuals’ intentions to comply with advised preventive measures [29]. Thus, this study expected a significant relationship between perceived severity and COVID-19 vaccine-uptake intention, and put forward the following hypothesis:

**H2.**  *Perceived severity is positively associated with behavioral intention to take the COVID-19 vaccine*.

***Perceived Benefits.*** In addition to the perceived threat of the illness, individuals’ expectations of the outcomes of performing healthy behaviors, i.e., perceived benefits, have been shown to impact individuals’ actions. In particular, a meta-analysis of HBM studies indicated that perceived benefit was the most powerful predictor among all the health belief factors [30]. Moreover, a study in the early stage of the COVID-19 pandemic revealed that the perceived benefits of preventative actions were positively associated with individuals’ intentions to take these actions [31]. Hence, we postulate the following hypothesis:

**H3.**  *Perceived benefits are positively associated with behavioral intention to take the COVID-19 vaccine*.

***Perceived Barriers.*** Meanwhile, perceived barriers will decrease the likelihood of protective behavior. Focusing on the negative aspects, perceived barriers refer to the tangible or psychological costs of adopting the preventative behavior. For instance, one of the barriers for females to get mammograms is that the process can be painful [32]. In the context of the COVID-19 vaccine, emerging information about the side effects and rising doubts about its effectiveness deterred people from taking the vaccine. Therefore, based on previous results regarding perceived barriers, we proposed the following hypothesis:

**H4.**  *Perceived barriers are negatively associated with behavioral intention to take the COVID-19 vaccine*.

***Cues to Action.*** Beyond the above health belief factors, individuals need to be activated by cues to adopt health actions [33]. As environmental triggers, cues to action could transfer psychological readiness into actual behavior [34,35]. Individuals obtain cues to action from various information sources, including print news, television shows, recommendations from health professionals and interpersonal interactions with peers [36,37,38].

Prior studies have demonstrated that cues are effective in increasing health behavior intentions. For instance, an HPV vaccine study reported that cues such as recommendations from friends or counseling from health professionals could boost HPV vaccination among women [39]. Another experimental study pointed out that cues in the form of health news could improve behavioral intentions as well [40]. In view of these studies, we expected the positive role of cues to action in the context of COVID-19 vaccination, and thus put forward the following hypothesis:

**H5.**  *Cues to action are positively associated with behavioral intention to take the COVID-19 vaccine*.

### 2.2. Linking Information Acquisition to the Health Belief Model

The acquisition of health information about COVID-19 is critical to help the public prevent infection. A significant body of literature on media effects has shown that communication factors such as media attention or interpersonal interactions influence individuals in an indirect manner rather than exerting direct effects [41,42]. According to the two-step process model, communication interventions alter individuals’ cognitive beliefs, which subsequently lead to attitudinal or behavioral changes [43]. As such, we assume that individuals’ acquisition of information on the COVID-19 vaccine would affect their intention to take the vaccine directly via mediators such as health beliefs. Thus, aside from examining the associations between health beliefs and intentions to take the COVID-19 vaccine, this study proposed to explore how information acquisition is linked to health beliefs regarding COVID-19 vaccination.

The rapid development of information and communication technology offers a growing multiplicity of available information sources for health-related information. For information on COVID-19, traditional media (e.g., printed newspaper, television), new media (e.g., social media, search engines, nongovernment websites) and interpersonal interactions have been significant sources of information [44]. These sources of information vary in terms of their accessibility, reliability, timeliness and accuracy [45,46]. Empirically, past studies on health risk communication have documented the distinctive roles of divergent information sources in promoting health behavior. For instance, traditional media performed superbly in shaping public attitudes and promoting health behavior changes due to its ability to disseminate large-scale information [47,48]. New media have been the most significant source for sharing information on public health crises [49,50,51]. Interpersonal interactions overshadow other information sources in terms of the social amplification of risk [52]. The studies presented thus far provided evidence that individuals’ reliance on different sources for COVID-19 vaccination information would significantly but differently influence their health beliefs about taking the vaccine.

Considering the disparate natures of information sources, the current study proposed to examine how individuals’ acquisition of COVID-19 vaccine information from different sources is related to their health belief factors, with a focus of inquiry on the role of traditional media, new media and interpersonal interactions. Given a dearth of research on these relationships, we postulated the following research questions:

RQ1: How does attention to traditional media about the COVID-19 vaccine relate to (a) perceived susceptibility, (b) the perceived severity of the COVID-19 pandemic, and (c) the perceived benefits, (d) perceived barriers and (e) cues to action of COVID-19 vaccination?

RQ2: How does attention to new media about the COVID-19 vaccine relate to (a) perceived susceptibility, (b) the perceived severity of the COVID-19 pandemic, and (c) the perceived benefits, (d) perceived barriers and (e) cues to action of COVID-19 vaccination?

RQ3: How do interpersonal interactions about the COVID-19 vaccine relate to (a) perceived susceptibility, (b) the perceived severity of the COVID-19 pandemic, and (c) the perceived benefits, (d) perceived barriers, and (e) cues to action of COVID-19 vaccination?

**Figure 1:**
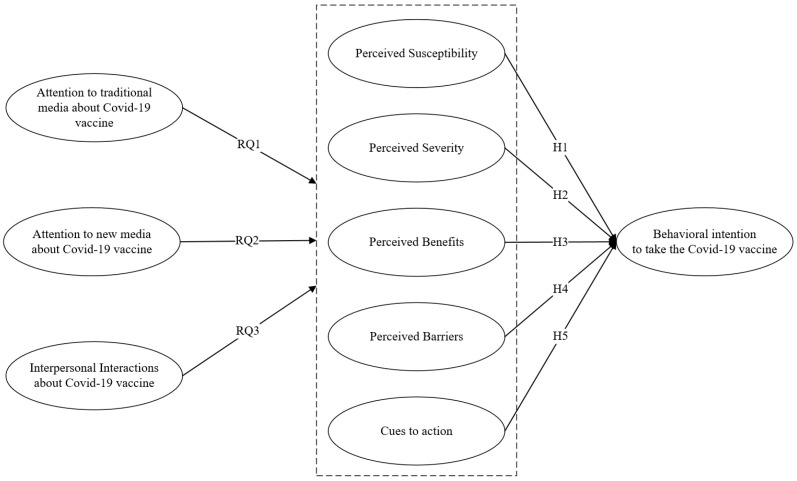
Hypothesized model.

The theoretical framework is displayed in Figure 1.

## 3. Methods

### 3.1. Data Collection

An online survey was conducted to collect data for this research during March 2021 in China. The web-based questionnaire was administered via a Chinese market research company, Wenjuanxing (https://www.wjx.cn/). Participants were recruited through the company, and a total of 697 adults completed questionnaires in this study. Two trap questions were included to filter out invalid respondents, which yielded a final sample of 621 respondents. We calculated the sample size based on the population size, the margin of error and the confidence level. Based on a margin of error of approximately ±4% at the 95% confidence level, a random sample of 601 respondents is required for a survey of a population of 1.413 billion. In this study, a total of 697 adults completed questionnaires. Based on the sample size calculator, a sample of 621 is needed for a representative study.

### 3.2. Measures

***Demographics.*** Demographic variables were used as control variables, including: age (Mdn = 27, M = 28.75, SD = 9.12), gender (56.4% of the respondents were female), education level (ranging from 1 = no formal education to 7 = PhD; Mdn = 5 or diploma or Bachelor degree; SD = 0.65) and monthly household income (ranging from 1 = CNY 3000 and below to 11 = above CNY 30,000; Mdn = 5 or CNY 9001 to 11,000; SD = 2.89).

***Attention to traditional media about the COVID-19 vaccine.*** Individuals’ attention to traditional media about the COVID-19 vaccine was measured using items adopted from previous studies [53,54]. The respondents were asked to rate how much attention (1 = no attention at all; 7 = very close attention) they paid to COVID-19 vaccine information provided by two media channels: newspapers (including print and digital editions) and television (M = 4.04, SD = 1.69, r = 0.575).

***Attention to new media about the COVID-19 vaccine.*** The measurement items for individuals’ attention to new media about the COVID-19 vaccine were adopted from a study by Besley and Shanahan [55]. The participants were asked to rate how much attention (1 = no attention at all; 7 = very close attention) they paid to COVID-19 vaccine information provided by two media channels: the internet and social media (M = 5.56, SD = 1.22, r = 0.565).

***Interpersonal interactions regarding the COVID-19 vaccine.*** Four modified items, adapted from prior research [56,57], were used to examine how frequently (1 = never; 7 = all the time) the respondents talked with (1) family, (2) friends, (3) co-workers and (4) health professionals about topics related to COVID-19 vaccination (M = 4.33, SD = 1.32, Cronbach’s α = 0.820).

***Perceived susceptibility.*** Three modified items adapted from prior research [37] were used to measure individuals’ perceived susceptibility to contracting COVID-19. The respondents were asked to indicate their levels of agreement on a 7-point Likert scale ranging from 1 (strongly disagree) to 7 (strongly agree) as follows: (1) The COVID-19 infection can happen to many people, including my family and friends; (2) I am at risk of contracting COVID-19; (3) COVID-19 infection can happen anytime to anyone, including a healthy individual (M = 4.68, SD = 1.58, Cronbach’s α = 0.849).

***Perceived severity.*** Five statements were adapted from previous studies to examine individuals’ perceived severity of COVID-19 infection [37,58,59]. On a 7-point Likert scale (1 = strongly disagree; 7 = strongly agree), respondents were asked to indicate their levels of agreement with the following statements: (1) COVID-19 is a fatal virus; (2) I believe that COVID-19 infection is extremely harmful; (3) I believe that COVID-19 infection will result in severe health problems; (4) I believe that COVID-19 infection will result in long-term health or economic sequelae; (5) I believe that COVID-19 infection has serious negative consequences (M = 5.47, SD = 1.24, Cronbach’s α = 0.892).

***Perceived benefits.*** Adapted from previous studies [37,59], four 7-point items were used to measure the respondents’ perceived benefits of taking the COVID-19 vaccine. Respondents were asked to indicate their agreement with the following statements: (1) Taking the COVID-19 vaccine will be effective in preventing me from contracting the virus; (2) taking the COVID-19 vaccine can help to boost my body immunity in battling the virus; (3) taking the COVID-19 vaccine can decrease the severity and the chance of having complications if people are infected; (4) taking the COVID-19 vaccine can prevent people from spreading the virus to others (M = 5.47, SD = 1.04, Cronbach’s α = 0.772).

***Perceived barriers.*** Adapted from Chen et al. [60], five 7-point items were used to measure respondents’ perceived barriers to taking the COVID-19 vaccine (1 = strongly disagree; 7 = strongly agree). The respondents were asked to indicate their agreement with the following statements on a scale from 1 (strongly disagree) to 7 (strongly agree): (1) I am generally opposed to vaccinations; (2) the COVID-19 vaccine has unpleasant side effects; (3) the COVID-19 vaccine can weaken the natural immune system; (4) taking the COVID-19 vaccine is inconvenient; (5) I am influenced by negative news about the COVID-19 vaccine (M = 2.84, SD = 1.24, Cronbach’s α = 0.847).

***Cues to action.*** To measure respondents’ cues to action regarding taking the COVID-19 vaccine, four modified items were adapted from prior research [59,60]. On a 7-point Likert scale, the respondents were asked to indicate their agreement with the following statements: (1) I would take a COVID-19 vaccine if health professionals recommend it, (2) I would take a COVID-19 vaccine if my families took it, (3) I would take a COVID-19 vaccine if my friends took it, and (4) I would take a COVID-19 vaccine if the media recommend it (M = 5.17, SD = 1.29, Cronbach’s α = 0.887).

***Behavioral intention to take COVID-19 vaccine.*** Three modified items adapted from previous research [37,59] were used to measure individuals’ intention to take the COVID-19 vaccine. The respondents were asked to indicate their agreement on a 7-point Likert scale ranging from 1 (strongly disagree) to 7 (strongly agree) as follows: (1) if the government will provide a free-of-charge COVID-19 vaccine within the next 12 months, I will receive it, (2) if I was given an opportunity to take a COVID-19 vaccine, I will receive it; (3) I plan to receive a COVID-19 vaccine within the next 12 months (M = 5.75, SD = 1.27, Cronbach’s α = 0.909).

### 3.3. Analytical Approach

Structural equation modeling (SEM) was used to test the hypothesized model using maximum likelihood estimation in *Mplus 7* [61]. Following a two-step analytical approach, we first performed a confirmatory factor analysis to estimate the measurement model, and then tested the structural equation model [62]. In the hypothesized model, age, gender, household income and education were included as control variables.

## 4. Results

Table 1 shows the statistics of all variables. Table 2 describes the bivariate correlations for relevant variables. The result of the measurement model indicated that all factor loadings were over 0.05 and mostly exceeded 0.80. The factor loadings for all the latent constructs were significant at the *p* < 0.001 level. Table 3 provides the descriptive statistics of the measurements and factor loadings for each latent variable in the model. The measurement model with nine latent constructs indicated a good model fit: χ^2^/(542) = 2.40, *p* < 0.001, CFI = 0.930, TLI = 0.919, RMSEA = 0.049. The hypothesized structural model was tested by controlling for age, gender, education and household income, which indicated an acceptable model fit: χ^2^/(515) = 2.33, *p* < 0.001, CFI = 0.935, TLI = 0.922, RMSEA = 0.047. Table 4 shows the results of the model fit indices.

Regarding RQ1, the results revealed that attention to traditional media about the COVID-19 vaccine was significantly related to individuals’ perceived severity of COVID-19 (*β* = 0.17, *p* < 0.05). However, such a significant relationship was not found between attention to traditional media and perceived susceptibility, perceived benefits, perceived barriers and cues to action regarding COVID-19 vaccination.

For RQ2, the results showed that attention to new media about the COVID-19 vaccine was positively related to perceived susceptibility (*β* = 0.17, *p* < 0.05), perceived benefits (*β* = 0.21, *p* < 0.01) and cues to action (*β* = 0.24, *p* < 0.01), and negatively associated with the perceived barriers (*β* = −0.48, *p* < 0.001). However, the analysis failed to reveal a significant relationship between attention to new media and perceived severity of COVID-19.

In terms of RQ3, which concerned the relationships between interpersonal interactions and health belief factors, the results unveiled that acquisition of information through interpersonal interactions was positively related to perceived severity (*β* = 0.18, *p* < 0.01), perceived benefits (*β* = 0.38, *p* < 0.001), perceived barriers (*β* = 0.22, *p* < 0.01) and cues to action (*β* = 0.34, *p* < 0.001). However, interpersonal interactions about the COVID-19 vaccine were not significantly associated with perceived susceptibility to contracting COVID-19.

**Figure 2:**
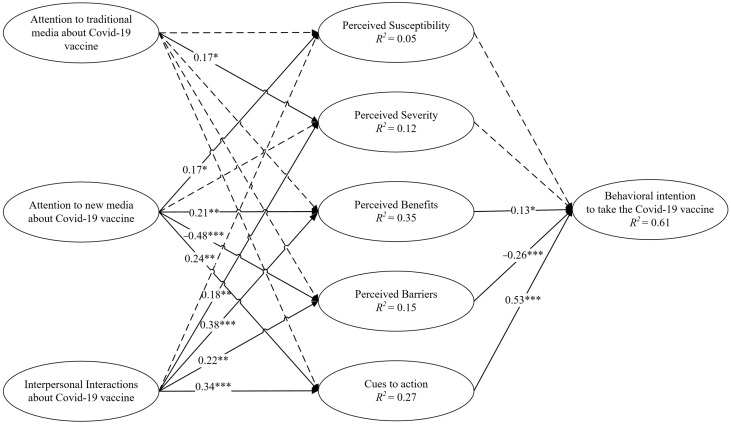
Structural equation model with standardized coefficients. Note: Dotted lines denote hypothesized nonsignificant paths. * *p* < 0.05, ** *p* < 0.01, *** *p* < 0.001.

Regarding the relationships between HBM constructs and behavioral intention, the results indicated that neither perceived susceptibility nor perceived severity of COVID-19 had a significant relationship with individuals’ intention to take the COVID-19 vaccine. Thus, H1 and H2 were not supported. Nonetheless, the perceived benefits of taking the vaccine were positively associated with vaccination intention (*β* = 0.13, *p* < 0.05), which supported H3. Likewise, perceived barriers were negatively associated with behavioral intention (*β* = −0.26, *p* < 0.001), supporting H4. Cues to action were also positively associated with individuals’ behavioral intention to take the COVID-19 vaccine (*β* = 0.53, *p* < 0.001), thereby supporting H5. Finally, the model explained 61.3% of the variance in behavioral intention to take the COVID-19 vaccine (see Figure 2), indicating that a set of correct variables was included.

Besides, we used bootstrapping to compare the estimates for the paths from different information sources to behavioral intention (see Table 5). The results revealed the advantageous role of new media in promoting vaccination intention, given the stronger standardized coefficient for the significant path from new media attention to behavioral intention to take the vaccine.

## 5. Discussion

Specifically, our findings on traditional media attention revealed a significant association with the perceived severity of COVID-19. This result is in line with previous studies indicating that attention to traditional media can help people make sense of the risk issue and form risk perceptions [63,64]. However, such significant relationships were not found for other health beliefs, including perceived susceptibility, perceived benefits, barriers and cues to action. We speculate that this might be due to the content broadcasted via traditional media during the COVID-19 outbreak. A content analysis study of COVID-19 reports revealed that traditional media coverage concentrated on macrolevel information of the pandemic’s development, such as updating the relevant statistics of infections and deaths [65], which would increase individuals’ perceived severity of COVID-19. However, people may find it difficult to relate macrolevel information to personal life, resulting in this media channel having no significant impacts on personally relevant factors such as perceived susceptibility, benefits, barriers and cues to action. This is in line with construal level theory, which suggests that people receiving macrolevel information tend to engage in high-level construal, which eliminates psychological distance and personal relevance [66].

An initial objective of the study was to identify the effective sources for disseminating information to promote COVID-19 vaccination. The most obvious finding to emerge from the analysis is that new media had more advantages in enhancing individuals’ health beliefs regarding vaccine uptake than traditional media and interpersonal interactions. Specifically, the findings suggested that attention to new media about COVID-19 vaccine was positively related to people’s perceived susceptibility, benefits and cues to action, but was negatively related to perceived barriers to taking the vaccine. This accords with previous studies which revealed the positive role of new media in increasing risk and benefit perceptions [67,68]. Moreover, public health risk studies have suggested that new media have been extensively used by public health agencies to communicate disease prevention information [69,70]. As new media have been the most accessed sources for COVID-19 information [71], it is unsurprising that the more attention that people paid to new media about the COVID-19 vaccine, the more health beliefs that they developed toward vaccination, which ultimately led to vaccination intentions.

Consistent with previous studies [37,72], this study also confirmed the important role of interpersonal interactions in promoting health behaviors. Specifically, the more people engaged in interpersonal discussions of the risks, the more perceived susceptibility they developed. During the COVID-19 outbreak, individuals were extensively exposed to interpersonal sources of health information (e.g., family, friends, colleagues, and health professionals), and such interpersonal communications may enhance risk perceptions, as social network members repeatedly exchange information on COVID-19 risks, infection rates and fatalities [73]. Moreover, our findings on the prominent role of interpersonal interactions as cues to action are in line with prior research, which showed that individuals’ vaccination intentions were largely influenced by interpersonal communications even more than media sources [37,73]. Besides, it is worth discussing the interesting facts revealed by the result that interpersonal interactions were positively related to both the perceived benefits of and barriers to taking the COVID-19 vaccine. A possible explanation for these results may be that people have conversations with their network members on both the benefits of and barriers to taking the vaccine [10,74]. In the case of COVID-19, individuals’ uncertainties about the effects of vaccine could make interpersonal interactions particularly influential, and this may explain the compelling impact of interpersonal interactions on the perceived benefits of and barriers to taking the vaccine.

Finally, this study confirmed the role of perceived benefits and barriers, and cues to action in promoting vaccination intentions, whereas the findings did not support previous research regarding the significant effects of perceived susceptibility and severity on health behaviors. This discrepancy could be attributed to individuals’ changing emotional reactions, as the pandemic was well contained in China. A longitudinal study tracking Chinese emotional reactions to the COVID-19 pandemic revealed that individuals’ risk perception decreased significantly over the four waves of data collection from February 2020 to January 2021 [75]. As the current study was conducted in China during early 2021, it is therefore likely that the respondents in our study had low levels of perceived severity and susceptibility to COVID-19. Thus, perceived severity and susceptibility would not be the primary factors to consider for vaccination, which resulted in the nonsignificant relationships between perceived severity and susceptibility, and intention to take the COVID-19 vaccine. Additionally, while typical vaccine development takes upwards of 10 years, COVID-19 vaccines were developed in less than a year after the outbreak [76]. Uncertainties about the safety and effectiveness of the vaccine drove people to ponder the benefits and barriers of taking the vaccine, which explained the significant relationships between the perceived benefits and barriers, and intentions. Individuals might turn to others for more cues to action, such as asking for suggestions from health professionals and people who had already taken the vaccine.

## 6. Implications and Limitations

Overall, this study has several theoretical and practical implications for the relevant domain. First, this study will fill the void in empirical research on the contributions of divergent information sources to promoting health behavior through affecting health beliefs. Secondly, this study offers insights into the literature on the HBM and health communication. Particularly, our findings contribute to the theoretical literature on the HBM by identifying media attention as the antecedent of health beliefs, suggesting that more attention should be paid to the factors affecting health beliefs aside from examining how health beliefs are related to health behaviors. Finally, findings from this study can help authorities and health professionals in identifying the most effective sources for disseminating health information during outbreaks of infectious diseases, as well as providing lessons for future vaccine programs. Practically, our findings on the significant effects of attention to new media over traditional media in promoting vaccination highlight the need for more utilization of new media in health communication. Despite the positive effects on the perceived benefits of vaccination, interpersonal interactions could increase individuals’ perceived barriers to taking the vaccine. Thus, the authorities should be cautious about the potential negative effects of interpersonal interactions on vaccine programs. Besides, future vaccine programs should pay more attention to public perceptions of the benefits of and barriers to taking a vaccine, given their significant effects on vaccination intentions. Additionally, the findings from this study could be helpful for authorities in regions with low vaccine acceptance rates.

Despite these findings and implications, the limitations of this study should be noted. First, this study failed to examine the effect of particular media platforms on vaccination. Taking new media for example, there are multiple new media platforms available through which the public can acquire information about COVID-19 pandemic, such as WeChat, Weibo, the quiz community and authorized websites. Moreover, individuals’ interactions with family, friends and health professionals may exert distinct effects as well. Thus, future research should pay detailed attention to different media platforms and social network members. Second, one of the promising future lines of research is to explore other factors such as vaccine type or sociodemographic factors, as suggested by prior vaccine hesitancy research [77]. Third, the convenience sample acquired from an internet-based survey may limit the generalizability of our findings. In particular, compared with the general Chinese population, our sample is younger. Different age cohorts may have different health beliefs regarding vaccines. The ways they make sense of and process health information may also be contingent upon age differences. Hence, additional examinations including respondents from diverse age cohorts are needed to better understand the effects of diverse information sources on promoting vaccination intentions. A larger sample size would improve the representativeness of the findings. Another limitation of this study was that we failed to examine the influence of other vaccination practices on individuals’ intention to take the vaccine, such as the implementation of the green pass that has been identified as an influential factor [78]. Finally, causality in the hypothesized model can only be inferred, due to the cross-sectional nature of the data in the current study. Future studies should address these limitations for development of the theory and practical contributions.

## 7. Conclusions

Overall, on the general basis of the HBM, this study examined how individuals’ acquisition of information on the COVID-19 vaccine from different sources was related to their vaccination intentions, highlighting an indirect communication process via health beliefs. In line with previous studies which applied the HBM to predict various health behaviors [16,79,80], our results suggested the significant role of perceived benefits, perceived barriers and cues to action in promoting intention to take the COVID-19 vaccine. More importantly, the findings revealed the differences in the effects of acquiring information via multiple sources among traditional media, new media, and interpersonal interactions. Notably, new media and interpersonal interactions were more salient in promoting vaccination intention via health beliefs, compared with traditional media. This suggests that in vaccine programs for public health crises such as the COVID-19 pandemic, it is necessary to disseminate information through new media and interpersonal interactions.

## Figures and Tables

**Table 1:** Summary of the statistics of all variables (*n* = 621).

	*n*	Mean	Median	Std. Deviation	Minimum	Maximum	Range
Age	621	28.75	27.00	9.12	18.00	75.00	18–57
Gender	621	1.56	2.00	0.50	1.00	2.00	1–2
Education	621	4.97	5.00	0.65	1.00	8.00	1–8
Income	621	4.99	5.00	2.89	1.00	11.00	1–11
Traditional Media Attention	621	4.04	4.00	1.69	1.00	7.00	1–7
New Media Attention	621	5.65	6.00	1.22	1.00	7.00	1–7
Interpersonal Interactions	621	4.33	4.25	1.32	1.00	7.00	1–7
Perceived Susceptibility	621	4.68	4.67	1.58	1.00	7.00	1–7
Perceived Severity	621	5.47	5.80	1.24	1.00	7.00	1–7
Perceived Benefits	621	5.47	5.50	1.04	1.50	7.00	1.5–7
Perceived Barriers	621	2.84	2.60	1.24	1.00	7.00	1–7
Cues to Action	621	5.17	5.25	1.29	1.00	7.00	1–7
Behavioral Intention	621	5.75	6.00	1.27	1.00	7.00	1–7

**Table 2:** Bivariate correlations among all variables (*n = 621*).

	(a)	(b)	(d)	(e)	(f)	(g)	(h)	(i)	(j)	(k)	(l)	(m)
(a) Age	1											
(b) Gender	−0.170 ***	1										
(c) Education	−0.210 ***	0.031	1									
(d) Income	0.091 *	−0.184 ***	0.212 ***	1								
(e) Traditional Media Attention	0.244 ***	−0.058	−0.127 **	0.123 ***	1							
(f) New Media Attention	0.075	0.030	−0.002	0.097 *	0.454 ***	1						
(g) Interpersonal Interactions	0.240 ***	−0.004	−0.062	0.087 *	0.526 ***	0.437 ***	1					
(h) Perceived Susceptibility	0.189 ***	−0.049	−0.048	0.017	0.207 ***	0.155 ***	0.242 ***	1				
(i) Perceived Severity	0.027	0.037	0.042	−0.007	0.102 *	0.183 ***	0.163 ***	0.241 ***	1			
(j) Perceived Benefits	0.228 ***	−0.054	−0.103 *	0.053	0.326 ***	0.326 ***	0.414 ***	0.382 ***	0.255 ***	1		
(k) Perceived Barriers	−0.012	−0.114 **	0.016	0.027	−0.063	−0.220 ***	−0.011	−0.001	0.066	−0.241 ***	1	
(l) Cues to Action	0.067	−0.005	−0.046	−0.019	0.297 ***	0.349 ***	0.411 ***	0.264 **	0.223 **	0.497 ***	−0.290 ***	1
(m) Behavioral Intention	0.054	0.037	−0.043	−0.113 **	0.237 ***	0.282 ***	0.319 ***	0.170 ***	0.172 ***	0.387 ***	−0.405 ***	0.653 ***

Note: * *p* < 0.05; ** *p* < 0.01; *** *p* < 0.001.

**Table 3:** Summary of measurement items (*n = 621*).

Variable	Loading	M	SD	AVE	CR
Traditional Media Attention (r = 0.58)				0.58	0.73
tma1	0.66	3.47	1.97		
tma2	0.85	4.62	1.84		
New Media Attention (r = 0.57)				0.57	0.73
nma1	0.79	5.67	1.38		
nma2	0.72	5.63	1.38		
Interpersonal Interactions (α = 0.82)				0.54	0.82
ii1	0.8	4.62	1.58		
ii2	0.77	4.74	1.44		
ii3	0.66	4.44	1.54		
ii4	0.69	3.52	1.97		
Perceived Susceptibility (α = 0.85)				0.64	0.84
psu1	0.96	4.55	1.90		
psu2	0.80	4.33	1.86		
psu3	0.60	5.16	1.64		
Perceived Severity (α = 0.89)				0.63	0.89
pse1	0.68	4.88	1.72		
pse2	0.87	5.64	1.43		
pse3	0.83	5.65	1.44		
pse4	0.89	5.75	1.32		
pse5	0.68	5.44	1.47		
Perceived Benefits (α = 0.77)				0.48	0.78
pbe1	0.81	5.61	1.17		
pbe2	0.78	5.78	1.14		
pbe3	0.57	5.26	1.40		
pbe4	0.56	5.21	1.63		
Perceived Barriers (α = 0.85)				0.52	0.84
pba1	0.76	2.12	1.48		
pba2	0.77	2.94	1.47		
pba3	0.84	2.57	1.49		
pba4	0.63	2.94	1.67		
pba5	0.58	3.61	1.74		
Cues to Action (α = 0.89)				0.61	0.86
cta1	0.75	5.48	1.36		
cta2	0.79	5.38	1.51		
cta3	0.81	5.11	1.49		
cta4	0.76	4.71	1.59		
Behavioral Intention (α = 0.91)				0.77	0.91
bi1	0.88	5.81	1.31		
bi2	0.90	5.78	1.34		
bi3	0.86	5.67	1.50		

Note: For reference, items appear in this table in the same order as in the Section 3.

**Table 4:** Measurement and structural model fit indices.

Model	χ^2^	df	χ^2^/df	CFI	TLI	RMSEA
Measurement	1301.78	542	2.40	0.930	0.919	0.049
HBM	1200.41	515	2.33	0.935	0.922	0.047

**Table 5:** The estimates for main paths in the hypothesized model (*n* = 621).

Path	Estimates	95% CI		*p*-Value
		LL	UL	
Traditional media attention → vaccine intention	0.003	−0.118	0.115	0.968
New media attention → vaccine intention	0.284	0.159	0.412	0.000
Interpersonal interactions → vaccine intention	0.176	0.077	0.275	0.004

Note: CI = confidence interval; LL = lower limit; UL = upper limit.

## Data Availability

Not applicable.
